# Capillary blood, overcoming dinosaur and unicorn stories

**DOI:** 10.1515/almed-2022-0115

**Published:** 2022-12-19

**Authors:** Álvaro González, Julia Maroto-García, Nerea Varo

**Affiliations:** Departamento de Bioquímica, Clínica Universidad de Navarra, Pamplona, Spain; Navarra Institute for Health Research (IdiSNA), Pamplona, Spain

**Keywords:** capillary blood, preanalytics, telemedicine

Senior specialists will remember the IVY method, or bleeding time test, a method used in the early 20th century to evaluate primary hemostasis. At present, this method sleeps in old books, as ancient and antique as dinosaurs. Nowadays, dried blood spot (DBS) analysis is a widely known method used in clinical laboratories worldwide, mainly in neonatal screening and in pharmacokynetic, toxicological and infectious disease studies. By this method, some drops of blood (some microliters) are drawn from the heel or finger via a relatively painless puncture and placed onto a designated filter paper. With this simple method, the blood sample is easily shipped to the laboratory, which makes this technique very adequate for neonatal screening. The World Health Organization recommends the use of DBS for screening for infectious diseases such as hepatitis B, C, and human immunodeficiency virus (HIV) in countries with deficient healthcare services [1]. However, although the majority of blood samples – virtually all liquid – are analyzed in the central laboratory, the use of DBS is anecdotal.

The chief executive officer (CEO) of Theranos, a promising blood testing start-up, was recently convicted of fraud for duping investors about the effectiveness of a technology her company had supposedly developed, which could detect multiple diseases from a few drops of capillary blood [2]. The history of this company, which was initially considered a unicorn in Silicon Valley, and her protagonist, an entepreneur that used to be compared with Steve Jobs, had all the ingredients of a thriller. This history teaches investors and laboratories that promises not based on scientific evidence should be approached with caution. However, Theranos technology pointed to one of the most compelling needs in healthcare: developing a blood sampling method that requires a minimal volume of blood and can be used out of the hospital.

Capillary blood sampling is a relatively painless procedure whereby a small volume of blood can be drawn by the patient. Various companies from different countries, including Spain, offer home-collect biochemistry kits for the analysis of capillary blood that include blood collection and shipping material. At-home blood collection would be extremely useful for patients living in remote areas far from blood collection centers, and could also complement the work of clinicians through the use of telemedicine. As a result of the pandemic, an increasing number of patients are reluctant to visit hospitals. These centers are frequently saturated, and remote blood sampling would alleviate the workload of laboratory collection centers. The use of small amounts of blood would clearly benefit some groups of patients, including pediatric, elderly, and chronic and cancer patients, who need frequent blood tests. Some patients are afraid of blood draws, and would show better tolerance to a less aggressive technique. However, this technique may not be recommended for some types of patients, including patients with circulatory problems, inflammation in the puncture site or coagulation problems.

Laboratory medicine specialists should approach these needs with an evidence-based rigorous approach. The preanalytical phase is critical and, although simple, needs to be adequately completed to ensure that the analytical process is robust, with acceptable variability. Methodological guides, such as CLSI GP42-ED7 of 2020 [3], should be adapted for inexperienced operators to be able to complete the process. There is a variety of self-sampling kits available in the market that allow to draw small amounts of capillary blood using lancets or more sophisticated devices such as those developed by Yourbio Health^®^ or Tasso^®^. These systems are designed to provide an adequate sample of blood and avoid heterogeneity, especially as a result of contamination with interstitial or intracellular fluid during finger “milking”. Inadequate handling may also cause hemolysis. There are tubes with a maximum volume of 1 mL for the collection of capillary blood, similar to common-use pediatric tubes, which could be directly used in laboratory autoanalyzers. As samples are collected out of the laboratory and shipping may take more than 24 h, it is critical that sample traceability and analyte stability are guaranteed during transportation. Shipping, which is generally provided by transport companies, should comply with the laws and regulations on the delivery of potentially hazardous biological materials.

Once in the laboratory, sample centrifugation and handling should be performed as in small samples such as pediatric samples. Sample quality indicators, including hemolysis and lipemia indices are necessary but not enough to determine sample heterogeneity. For the use of capillary blood to become commonplace, the autoanalyzers and reagents used in central laboratories should be adapted to small volumes of blood. One of the main drawbacks is the high dead volume of equipments, which frequently reaches 100 µL, adapted to small samples of venous blood. When capillary blood samples are used, which usually contain less than a hundred of microliters, dead volume should be minimal or, ideally, none. In addition, capillary blood has different characteristics and composition, as compared to venous blood, since it is a combination of blood from arterioles, capillary veins and venules. Of note, capillary blood may have different analyte concentrations than venous blood. Hence, different reference values should be used for clinical interpretation. Additionally, reagents are generally designed for use in venous blood, and comparative studies against capillary blood are required. Some studies have demonstrated an excellent correlation for some analytes, such as glycated hemoglobin [4].

The simplicity of the collection method and the small amount of blood needed for the analysis of liquid samples of capillary blood render it a promising technique that will progressively gain ground over venous blood in the clinical laboratory. Some of the challenges that laboratories will face in the coming years include improving pre-analytical control, ensuring sample traceability, and validating analytical methods. The use of capillary blood samples will afford the opportunity to provide top-quality services to patients, clinicians and health systems, thereby allowing us to leave dinosaur ideas and unicorn visions behind.
